# Lung cancer screening by single-shot dual-energy subtraction using flat-panel detector

**DOI:** 10.1007/s11604-021-01163-z

**Published:** 2021-06-26

**Authors:** Hiroshi Mogami, Yumiko Onoike, Hiroshi Miyano, Kenji Arakawa, Hiromi Inoue, Kouji Sakae, Toshiaki Kawakami

**Affiliations:** Ehime General Healthcare Association, 1-10-5, Misake-cho, Matsuyama, Ehime 790-0814 Japan

**Keywords:** Lung cancer, Chest radiograph, Energy subtraction, Lung cancer screening, Flat-panel detector

## Abstract

**Purpose:**

The purpose of this study was to evaluate the usefulness of single-shot dual-energy subtraction (DES) method using a flat-panel detector for lung cancer screening

**Materials and methods:**

The subjects were 13,315 residents (5801 males and 7514 females) aged 50 years or older (50–97 years, with an intermediate value of 68 years) who underwent lung cancer screening for a period of 1 year and 6 months from January 2019 to June 2020. We investigated whether the number of lung cancers detected, the detection rate, and the rate of required scrutiny changed, when DES images were added to the judgment based on conventional chest radiography.

**Results:**

When DES images were added, the number and percentage of cancer detection increased from 16 (0.12%) to 23 (0.17%) (*P* < 0.05). Five of the newly detected 7 lung cancers were in the early stages of resectable cancer. The rate of participants requiring scrutiny increased slightly from 1.1 to 1.3%.

**Conclusion:**

DES method improved the detection of lung cancer in screening. The increase in the percentage of participants requiring scrutiny was negligible.

## Introduction

The effectiveness of lung cancer screening by chest radiography is considered to be limited [1], and some researches on lung cancer screening by low-dose CT are ongoing [2, 3]. The detection of lung cancer by chest radiography is not sufficient and the detection rate has been reported to be 70–80% [4, 5]. This is mostly due to the fact that the ribs and clavicles overlap the lungs on chest radiography, making it difficult to detect nodular shadows [6]. Recently, several methods have been developed to remove bones from chest radiography. One is software-based bone suppression (BS) method [7]. The other is DES method, which removes bone by acquiring two images with different energies and subtracting them [8–10]. Of these, one is a single-exposure method using storage phosphor system and the other is a double-exposure method using an FPD. The former has problems with image quality and processing performance [11], while the latter is not widely used due to problems such as increased radiation dose and motion artifacts caused by double-exposure [10, 12]. In the present study, we performed lung cancer screening using a newly developed system that can generate DES images by a single-exposure method with two overlapping FPDs and verified the effectiveness of the system.

## Materials

This study was conducted on residents who underwent lung cancer screening for a period of 1 year and 6 months from January 2019 to June 2020. Chest X-rays were taken on site using a health screening bus. A total of 13,315 residents (5801 males and 7514 females) aged 50 years and older (50–97 years, intermediate age 68 years) with or without smoking were included in the study.

## Methods

The generator was Radnext CS (Hitachi Medico, Tokyo, Japan). The X-ray receiving system was CALNEO Dual (Fujifilm, Tokyo, Japan). The tube voltage was 130 kVp, the tube current was 80 mA, and automatic imaging system was performed using a photo timer. Based on the phantom experiment, the timer was extended by about 20% longer than usual to ensure the signal-to-noise ratio and graininess of DES images. The surface dose without DES was 0.210 mGy at 130 kV, 2.4 mAs, while with DES it was 0.252 mGy at 130 kV, 2.8 mAs. These were measured with the application software, Sdec v6. The National diagnostic reference levels in Japan (2020) are 0.30 mGy for frontal chest radiography, so both values are within that range. The panel size was 46 × 46 cm, and the spatial resolution was 5 pixels/mm, corresponding to 2300 × 2300 pixels per image. The distance between the tube and the subject was 200 cm. The FPD places CsI in the foreground to produce conventional chest images and GOS in the background to produce high-energy images. The high-energy image was emphasized, weighted and subtracted to produce a soft tissue image and a bone image. After exposure, it took about 7 s for DES images to be generated, and DES images and conventional images were displayed on the monitor in turn for image confirmation. There was no burden on the radiology technician, and the participants did not have to wait. For reading, we used a viewer with two 5 M monitors (ScrEagle, Miura, Hiroshima, Japan) for observation and inputting judgments: one for the current image and the other for the past image.

**Fig. 1:**
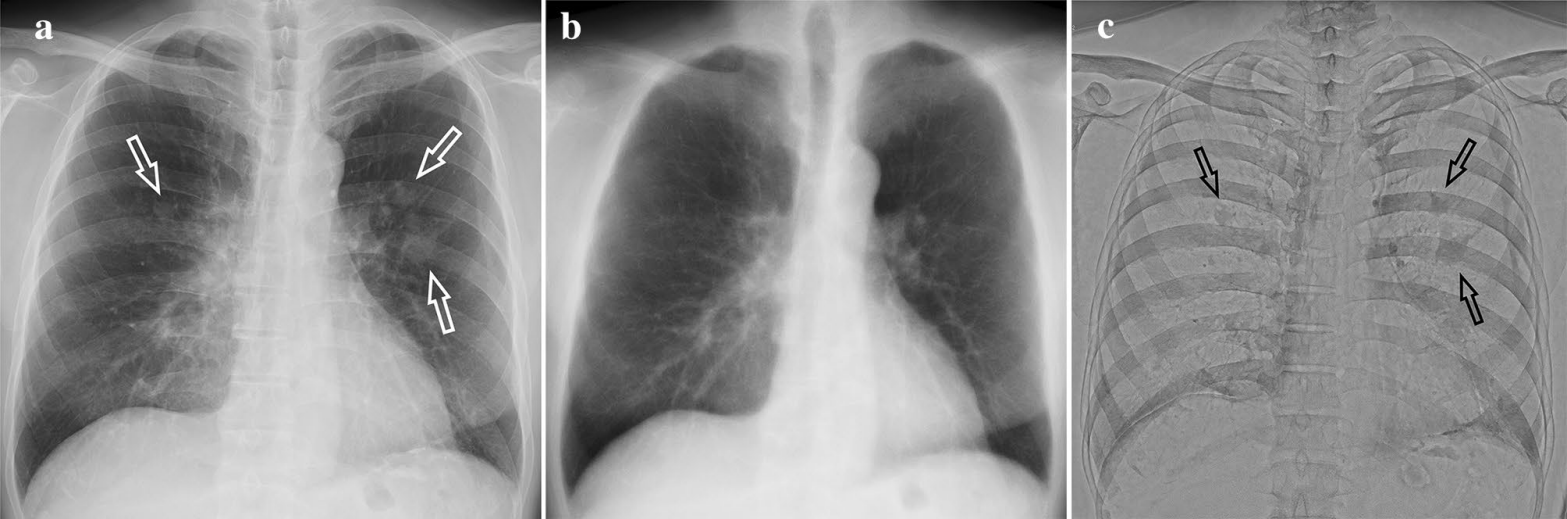
Male, 80 s, pleural calcifications. **a** Conventional radiography showed bilateral nodular shadows (arrows). **b** The soft tissue image showed no nodular shadows. **c** The bone image showed bilateral nodular shadows(arrows), which were determined to be pleural calcifications

The reading experiment was conducted by a single reader. For double reading, another readers read the chest radiography without DES images. This decision was not used in this experiment. First, only the conventional image was read and judged. Next, on a different day (3 days or less), the conventional image was read with DES images added and judged blindly. DES images consisted of soft tissue images with the bone removed and bone images with the soft tissue removed. Conventional images, soft tissue images, and bone images were read in this order. In the observation of soft tissue images, there was no obvious criterion for determining that the lesion required scrutiny, but if a region of interest was found, the current and past images were reviewed carefully to determine if it was a new lesion or not. If any changes were observed over time, the lesion required scrutiny. Findings of merely faint increased attenuation or less than 5 mm did not require scrutiny which were considered to be of little clinical significance. Abnormalities in the ribs and calcification of the lungs and pleura can be confused with lung cancer nodules by conventional imaging alone, but with DES method, they disappear on soft tissue images and appear on bone images, so we were able to confidently determine that there was no need for scrutiny (Fig. 1).

We examined whether the presence or absence of DES images changed the percentage of participants who required scrutiny and the number of cancers detected.

## Results

**Fig. 2:**
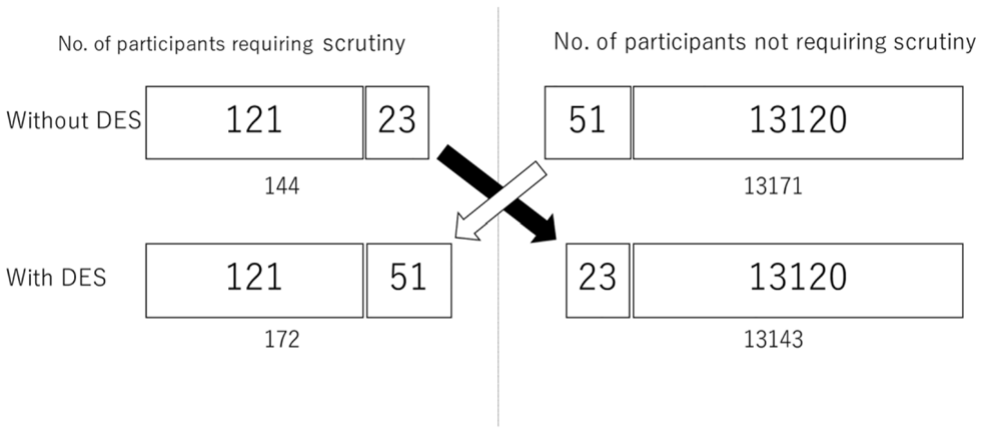
Change in number of the participants requiring scrutiny with and without DES images

There were 121 participants who required scrutiny in the absence of DES images and remained unchanged with the addition of DES images. There were 51 participants who did not require scrutiny in the absence of DES images but required it with the addition of DES images. There were 23 participants who required scrutiny in the absence of DES images but no longer required it with the addition of DES images (Table 1 and Fig. 2).

**Table 1:** Change in number of the participants requiring scrutiny with and without DES images

	DES −/ +	DES −/+	DES −/+
Scrutiny	pos → pos	neg → pos	pos → neg
Number	121	51	23

*DES −* without DES images, *DES +* with DES images, *pos* positive, *neg* negative

Finally, the percentage of participants requiring scrutiny increased slightly from 1.1 to 1.3% (Table 2). The number of cancers detected was 16 without DES images, but 7 new cancers were detected using DES images, bringing the total to 23. The cancer detection rate increased from 0.12 to 0.17% (Table 3). There was a statistically significant difference between the two groups by McNemar’s test (*P* < 0.05). Based on these results, the positive predictive value increased from 11 (16/144) to 13% (23/172) (n.s.).

**Table 2:** Final determination of scrutiny with and without DES images

	Percentage (%)	Number
DES −	1.1	144
DES +	1.3	172

*DES −* without DES images, *DES +* with DES images

**Table 3:** Percentage and number of newly detected lung cancers

	Percentage (%)	Number
DES −	0.12	16
DES +	0.17	23

*DES −* without DES images, *DES +* with DES images

**Fig. 3:**
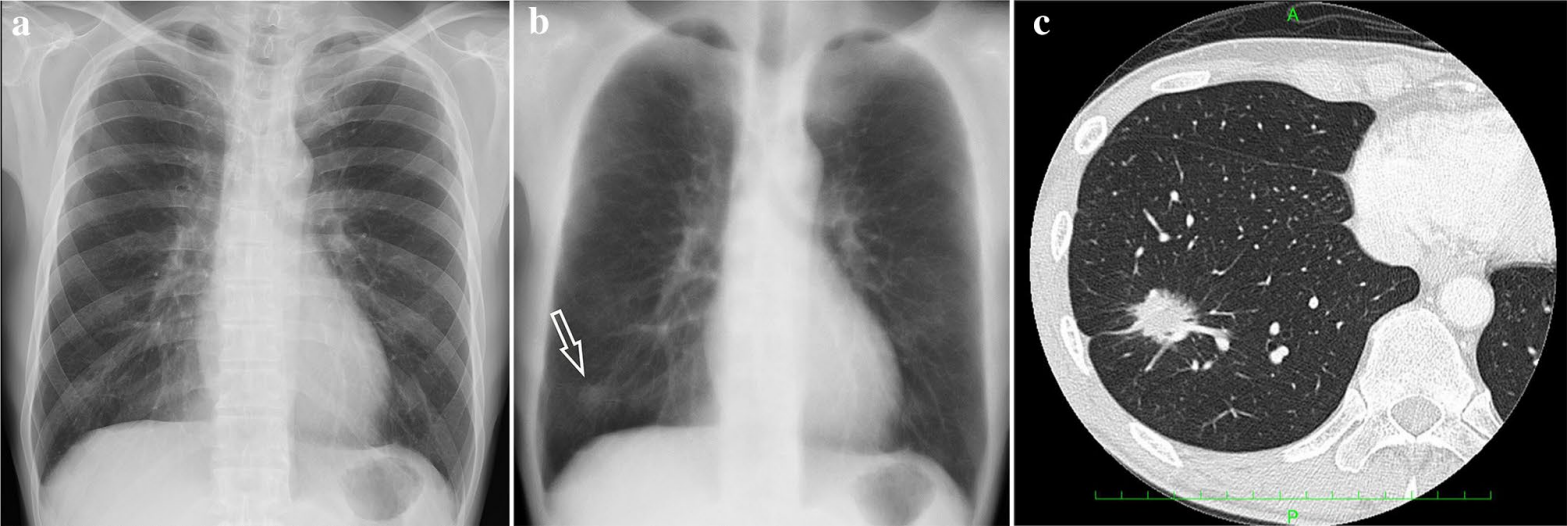
Male, 60 s, adenocarcinoma, 30 mm. **a** No abnormal findings were observed on conventional radiography. **b** The soft tissue image showed a nodular shadow in the right lower lung field (arrow). **c** CT showed a solid nodular shadow with GGA in the right lower lobe

**Fig. 4:**
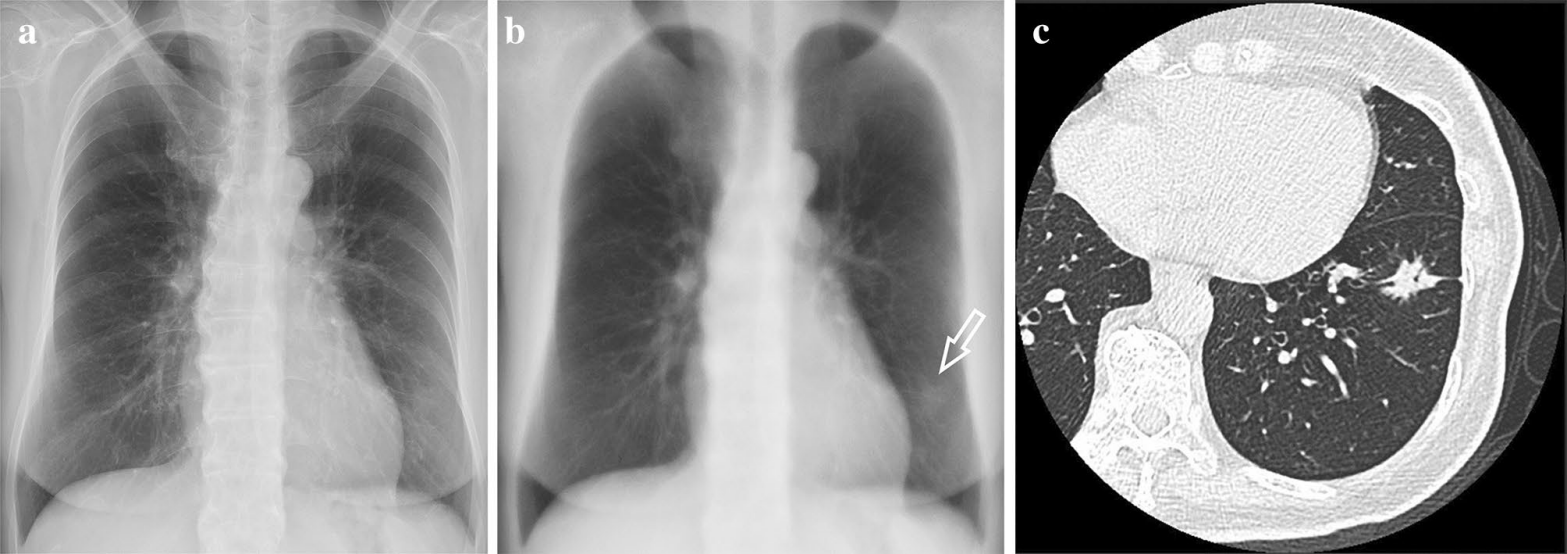
Female, 60 s, adenocarcinoma, 22 mm. **a** No abnormal findings were observed on conventional radiography. **b** The soft tissue image showed a nodular shadow in the left lower lung field (arrow). **c** CT showed a nodular shadow in the left lower lobe

**Fig. 5:**
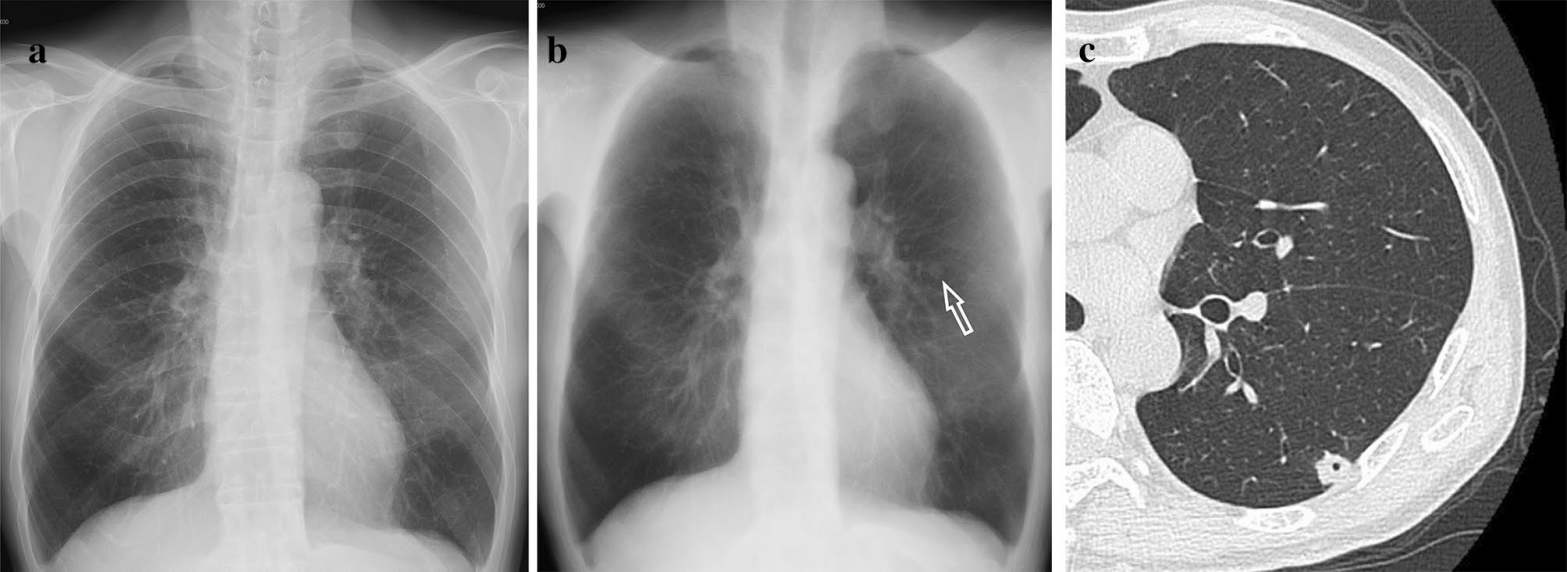
Male, 70 s, squamous cell carcinoma, 10 mm. **a** No abnormal findings were observed on conventional radiography. **b** The soft tissue image showed a nodular shadow in the left middle lung field (arrow). **c** CT showed a nodular shadow in the left lower lobe

There were five stage I patients, four of whom had adenocarcinoma and underwent resection, and one patient underwent radiotherapy without histological proof. Another patient had squamous cell carcinoma and was followed up for 4 months without histological proof, and was treated with chemotherapy because of tumor growth and metastasis. The other patient had lung metastasis from breast cancer. (Table 4 and Figs. 3, 4 and 5).

**Table 4:** Newly detected lung cancers with DES images

Age/gender	Histology	Size (mm)	Stage	Therapy
60 s/M	Adenoca	30	IB	Ope
80 s/F	Adenoca	30	IA1	Ope
80 s/M	Adenoca	30	IA3	Ope
60 s/F	Adenoca	22	IA2	Ope
80 s/M	Unknown	13	IA1	RT
70 s/M	Sq	10	IVA post F/U	CT
50 s/F	Mts	10		CT

*Adenoca* adenocarcinoma, *Sq* squamous cell carcinoma, *F/U* follow up, *Ope* operation, *RT* radiation therapy, *CT* chemotherapy, *Mts* metastasis

## Discussion

Recently, methods to remove bone from chest radiography has been developed, and several reading experiments have been reported to investigate the detection rate of lung nodules [10–13]. This is the first report of the use of DES method for screening of lung cancer. With the addition of DES images, new lung cancers were detected. Most of the newly detected lung cancers were resectable early-stage cancers. With the addition of DES images, the detection rate of lung cancer has improved by 30%, from 0.11 to 0.17%. However, it was not higher than that of CT. On the other hand, among the lung cancers detected by CT, there were not a few cases of adenocarcinoma in situ and minimally invasive adenocarcinoma, mainly GGA, and the problem of overdiagnosis has been proposed [14]. In the present study, no pure GGA lesions were found. Even with the bone removed, it was not easy to pick up the slight attenuation differences in the GGA lesions on the DES images, which depicted thick, overlapping lung structures on a single 2D image. However, in previous reports [15, 16], adenocarcinoma of part-solid type on CT was a useful target for DES method, and the newly discovered cancer in this study included such lesions. In other words, DES images has the potential to detect life-threatening lung cancer at a time when it is resectable, and if used for lung cancer screening, it is expected to reduce the number of lung cancer deaths.

In soft tissue images, the removal of the ribs made the contours and attenuation differences of the lung cancer nodules much clearer. The X-ray receiving system used in this study was a FPD which has a higher DQE than the system using the storage phosphor system [17, 18]. In addition, advances in image processing have improved the signal-to-noise ratio over the entire lung field. The radiation dose was only about 20% higher than that of modern FPDs because of the single-shot nature of the system. The dose is markedly lower than that of the film-screen method, the storage phosphor system or double-shot energy-subtraction system.

The rate of participants requiring scrutiny increased only slightly from 1.1 to 1.3%. The number of participants requiring scrutiny increased by 51 with the addition of soft tissue images. However, this was offset by a decrease of 23 participants due to the observation of bone images. Unlike BS method, which attempts to remove bone by software image processing, DES images are generated from two different energy images and are material discriminating. Therefore, a lesion that disappears in soft tissue images and shows up in bone images is unambiguously a bone or calcified lesion and is excluded from cancer candidacy. This is the advantage over BS method, and it is expected to reduce the rate of requiring scrutiny as much as possible in the screening.

A limitation of this study is that there was only one reader in this experiment, whereas the 2-month pilot study used multiple readers. Since there was a large difference in the judgments of the readers, it was decided to use one reader in this experiment. In this paper, we were able to show the effectiveness of DES method in actual clinical practice under limited conditions, but we were not able to sufficiently verify its universality. We are now waiting for a multi-center validation.

## Conclusion

DES method improved the detection of lung cancer in screening. The increase in the percentage of participants requiring scrutiny was negligible. DES method also minimized the increase in radiation exposure.

## Abbreviations

CT: Computed tomography
FPD: Flat-panel detector
GGA: Ground-glass attenuation
DQE: Detective quantum efficiency
